# Manometric and pH-monitoring changes after laparoscopic sleeve gastrectomy: a systematic review

**DOI:** 10.1007/s00423-021-02171-3

**Published:** 2021-04-14

**Authors:** Andrea Balla, Francesca Meoli, Livia Palmieri, Diletta Corallino, Maria Carlotta Sacchi, Emanuela Ribichini, Diego Coletta, Annamaria Pronio, Danilo Badiali, Alessandro M. Paganini

**Affiliations:** 1 Bariatric Surgery Unit, Department of General Surgery and Surgical Specialties “Paride Stefanini”, Sapienza University of Rome, Azienda Policlinico Umberto I, Viale del Policlinico 155, 00161 Rome, Italy; 2 Department of Translational and Precision Medicine, Sapienza University of Rome, Azienda Policlinico Umberto I, Viale del Policlinico 155, 00161 Rome, Italy; 3 Department of General Surgery, Emergency Department, Emergency and Trauma Surgery Unit, Sapienza University of Rome, Azienda Policlinico Umberto I, Viale del Policlinico 155, 00161 Rome, Italy; 4 Digestive Endoscopy Unit, Department of General Surgery and Surgical Specialties “Paride Stefanini”, Sapienza University of Rome, Azienda Policlinico Umberto I, Viale del Policlinico 155, 00161 Rome, Italy

**Keywords:** Gastroesophageal reflux disease (GERD), Sleeve gastrectomy, Manometry, pH-metry, 24h-pH-impedance

## Abstract

**Purpose:**

Aim of this systematic review is to assess the changes in esophageal motility and acid exposure of the esophagus through esophageal manometry and 24-hours pH-monitoring before and after laparoscopic sleeve gastrectomy (LSG).

**Methods:**

Articles in which all patients included underwent manometry and/or 24-hours pH-metry or both, before and after LSG, were included. The search was carried out in the PubMed, Embase, Cochrane, and Web of Science databases, revealing overall 13,769 articles. Of these, 9702 were eliminated because they have been found more than once between the searches. Of the remaining 4067 articles, further 4030 were excluded after screening the title and abstract because they did not meet the inclusion criteria. Thirty-seven articles were fully analyzed, and of these, 21 further articles were excluded, finally including 16 articles.

**Results:**

Fourteen and twelve studies reported manometric and pH-metric data from 402 and 547 patients, respectively. At manometry, a decrease of the lower esophageal sphincter resting pressure after surgery was observed in six articles. At 24-hours pH-metry, a worsening of the DeMeester score and/or of the acid exposure time was observed in nine articles and the de novo gastroesophageal reflux disease (GERD) rate that ranged between 17.8 and 69%. A meta-analysis was not performed due to the heterogeneity of data.

**Conclusions:**

After LSG a worsening of GERD evaluated by instrumental exams was observed such as high prevalence of de novo GERD. However, to understand the clinical impact of LSG and the burden of GERD over time further long-term studies are necessary.

## Introduction

Laparoscopic sleeve gastrectomy (LSG) is presently considered a safe, easy, and effective stand-alone procedure for the treatment of morbid obesity, in terms of weight loss and comorbidity resolution and with low morbidity [1–5]. For these reasons, LSG quickly gained widespread popularity both in the USA and in Europe [5–8].

In obese patients with proven gastroesophageal reflux disease (GERD), the bariatric surgical treatment of choice is laparoscopic Roux-en-Y gastric bypass (LRYGB) due to its excellent results in terms of GERD resolution [9–11], but the outcomes of GERD after LSG are still debated [2–5]. Most published articles analyzing GERD after LSG investigate symptoms [1, 12], but several obese patients have few or no GERD symptoms, and often there is no agreement between the presence of symptoms and the diagnostic assessment findings [5, 8, 13]. For this reason, the introduction of endoscopy, manometry, and 24-hours pH-monitoring in the routine pre- and postoperative workup of patients candidate to bariatric surgery could change the management strategy in these patients [1–3, 5, 8, 13]. The detection of GERD by instrumental assessment in asymptomatic patients before surgery could switch the indication from LSG to LRYGB, while the postoperative diagnosis of severe GERD, albeit asymptomatic, could set the indication for re-do surgery from LSG to LRYGB, in order to prevent the occurrence of Barrett’s esophagus [1–3, 5, 8, 13].

Another debated issue about GERD in bariatric surgery is the presence of hiatal hernia [2, 3, 10, 12, 13]. Some authors reported an improvement in GERD symptoms when hiatal hernia is diagnosed and repaired, even if these data are not confirmed by others [2, 3, 10, 12, 13].

The aim of this systematic review is to assess the changes in esophageal motility and acid exposure of the esophagus through esophageal manometry and 24-hours pH-monitoring, before and after LSG.

## Materials and methods

Institutional review board approval and informed consent from participants were not needed for this systematic review.

### Inclusion criteria

Inclusion criteria were (1) articles from any country written in English, Spanish, or Italian and (2) articles in which all included patients underwent manometry and/or 24-hours pH-metry or both, before and after LSG.

### Exclusion criteria

Exclusion criteria were (1) assessment of gastroesophageal reflux only by patients’ reporting of symptoms and/or endoscopy; (2) bariatric surgical techniques different from LSG; (3) gastroesophageal reflux assessment performed only in selected patients; (4) evaluation by manometry or pH-metry performed only before or only after LSG; (5) presence of associated procedures (omentopexy); (6) reviews, systematic reviews, meta-analysis, studies with data retrieved from registries, comments, case reports, correspondence and letters to editor, editorials, technical surgical notes, and imaging studies; and (7) animals involvement.

### Search strategy

We conducted a systematic review of published articles according to the preferred reporting items for systematic review and meta-analysis (PRISMA) statement [14]. The search was carried out in the PubMed, Embase, Cochrane, and Web of Science databases [15] using the keywords reported in Table 1.

**Table 1:** Keywords used for research in the PubMed, Embase, Cochrane, and Web of Science databases

sleeve gastrectomy AND ph manometry	
sleeve gastrectomy AND ph-manometry	
bariatric surgery AND ph manometry	
bariatric surgery AND ph-manometry	
sleeve gastrectomy AND manometry	
bariatric surgery AND manometry	
sleeve gastrectomy AND reflux	
bariatric surgery AND reflux	
sleeve gastrectomy AND GERD	
bariatric surgery AND GERD	
sleeve gastrectomy AND 24h-pH-impedance	
sleeve gastrectomy AND 24h pH impedance	
sleeve gastrectomy AND pH-impedance	
sleeve gastrectomy AND pH impedance	
bariatric surgery AND 24h-pH-impedance	
bariatric surgery AND 24h pH impedance	
bariatric surgery AND pH-impedance	
bariatric surgery AND pH impedance	

Overall, the search revealed 13769 articles published until November 2019. Of these, 9702 were eliminated because they were found more than once. Of the remaining 4067 articles, further 4030 were excluded after screening title and abstract because they did not meet the inclusion criteria.

### Quality assessment of included articles

Two authors (A.B. and F.M.) assessed the quality of the included papers by a modified Newcastle-Ottawa Scale (NOS) for cohort studies [16]. NOS is based on three factors for the evaluation of each paper: patients’ selection, comparability, and the completeness of the reported results (postoperative outcomes) [16]. The maximum score attributable to each article is nine points [16].

### Risk of bias assessment of included articles

Two authors (A.B. and F.M.) assessed the risk of bias of the included articles by the Risk Of Bias In Non-randomized Studies of Interventions (ROBIN-I) tool [17]. ROBIN-I includes seven domains: the first two domains concern confounding and selection of participants into the study, the third domain addresses classification of the interventions, and the last four domains address biases due to deviations from intended interventions, missing data, outcomes measurement, and selection of the reported result [17]. Based on judgments assigned for each domain, at the end, one single judgment is assigned to the entire article [17].

### Study design

After screening the title and abstract, the articles that fulfilled the inclusion criteria were identified, and their full text was reviewed. Data were extracted and stored in the Microsoft Excel program (Microsoft Corporation, Redmond, Washington, USA).

The following data were extracted from each article: authors, year of publication, type of study, number of patients, gender, age, presence of hiatal hernia, bougie size, distance of gastrectomy from pylorus, if oversewing was performed, pre- and postoperative body mass index (BMI), manometry or 24-hours pH-metry technique employed, manometric and pH-metric data, symptoms, and timing of postoperative manometric, pH-metric, and symptom evaluation.

## Results

**Fig. 1:**
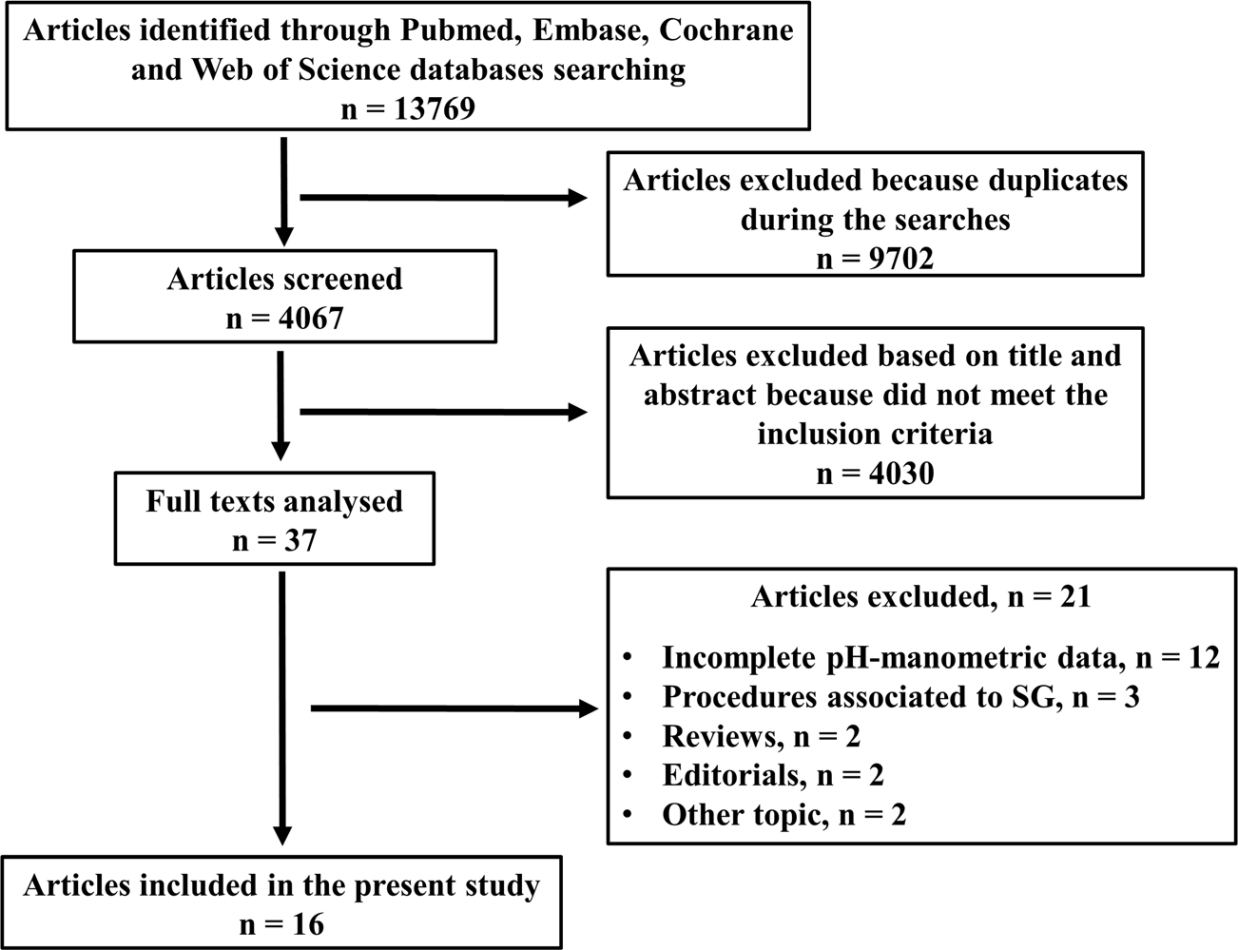
Preferred reporting items for systematic review and meta-analysis (PRISMA) flow diagram [14]. SG: sleeve gastrectomy

Thirty-seven articles were fully analyzed, and 21 further articles were excluded. Finally, 16 articles published between February 2013 and September 2019 were included [18–33], as shown in the PRISMA flow diagram (Fig. 1) [14]. The assessments of quality based on NOS and of the risk of bias based on ROBIN-I of the included articles are shown in Tables 2 and 3.

**Table 2:** Assessment of the articles quality based on the Newcastle-Ottawa scale (NOS) [16]

Author, year, type of study	Selection	Comparability	Outcomes	Total Score	Assessment
	1 2 3 4	5	6 7 8		
Braghetto, 2010, prospective [18]	- - * *	- -	* - *	4	Poor
Gorodner, 2015, prospective [19]	* - * *	- -	* * -	5	Poor
Valezi, 2017, prospective [20]	* * * *	* -	* * *	8	Good
De Angulo, 2019, prospective [21]	* - * *	- -	* * *	6	Poor
Sioka, 2017, prospective [22]	* - * *	- -	* * *	6	Poor
Rebecchi, 2014, prospective [23]	* - * *	- -	* * *	6	Poor
Kleidi, 2013, prospective [24]	* - * *	- -	* - *	5	Poor
Gemici, 2020, retrospective [25]	* - * *	- -	* - *	5	Poor
Coupaye, 2018, prospective [26]	- - * *	- -	* * *	5	Poor
Del Genio 2014, prospective [27]	* - * *	- -	* * *	6	Poor
Tolone, 2020, prospective [28]	* - * *	- *	* * *	7	Fair
Burgerhart, 2014, prospective [29]	* - * *	- -	* - *	5	Poor
Georgia, 2017, prospective [30]	* - * *	- -	* * *	6	Poor
Świdnicka-Siergiejko, 2018, prospective [31]	* - * *	- -	* * *	6	Poor
Thereaux, 2016, prospective [32]	* * * *	* *	* - *	8	Good
Yormaz, 2017, retrospective [33]	* * * *	* *	* * *	9	Good

**Table 3:** Assessment of risk of bias of the included articles based on Risk Of Bias In Non-randomized Studies of Interventions (ROBIN-I) [17]

Author, year, type of study	Bias due to confounding	Bias in selection participants	Bias in classification of interventions	Bias due to deviations from intended interventions	Bias due to missing data	Bias in measurement of outcomes	Bias in selection of reported result	Overall
Braghetto, 2010, prospective [18]	Serious	Low	Low	Low	Low	Moderate	Moderate	Serious
Gorodner, 2015, prospective [19]	Serious	Low	Low	Low	Low	Low	Low	Serious
Valezi, 2017, prospective [20]	Moderate	Low	Low	Low	Low	Low	Low	Moderate
De Angulo, 2019, prospective [21]	Serious	Low	Low	Low	Low	Low	Low	Serious
Sioka, 2017, prospective [22]	Serious	Low	Low	Low	Low	Low	Low	Serious
Rebecchi, 2014, prospective [23]	Serious	Low	Low	Low	Low	Low	Low	Serious
Kleidi, 2013, prospective [24]	Serious	Low	Low	Low	Low	Low	Low	Serious
Gemici, 2020, retrospective [25]	Serious	Low	Low	Low	Low	Low	Low	Serious
Coupaye, 2018, prospective [26]	Serious	Low	Low	Low	Low	Low	Low	Serious
Del Genio 2014, prospective [27]	Serious	Low	Low	Low	Low	Low	Low	Serious
Tolone, 2020, prospective [28]	Serious	Low	Low	Low	Low	Low	Low	Serious
Burgerhart, 2014, prospective [29]	Serious	Low	Low	Low	Moderate	Low	Low	Serious
Georgia, 2017, prospective [30]	Serious	Low	Low	Low	Low	Low	Low	Serious
Świdnicka-Siergiejko, 2018, prospective [31]	Serious	Low	Low	Low	Low	Low	Low	Serious
Thereaux, 2016, prospective [32]	Low	Low	Low	Low	Moderate	Moderate	Low	Moderate
Yormaz, 2017, retrospective [33]	Low	Low	Moderate	Low	Moderate	Moderate	Moderate	Moderate

Low: low risk of bias (the study is comparable to a randomized trial). Moderate: moderate risk of bias (the study provides sound evidence for a non-randomized study but cannot be considered comparable to a randomized trial). Serious: serious risk of bias (the study has important problems)

Manometric and pH-metric data were obtained from 402 and 547 patients, respectively [18–33]. Hiatal hernia was diagnosed preoperatively in 32 patients [19, 21, 23, 26, 29, 32], and in four cases it was repaired. In one patient by cruroplasty, even if it was not specified whether anterior or posterior [21], and in the other three cases, the type of hiatal repair was not specified [32] (Table 4). Overall, 29 hiatal hernias were diagnosed postoperatively. A bougie size of 36 French (Fr) was used in eight studies (417 patients) [19, 21–23, 25, 26, 32, 33], a bougie of 32 Fr was used in two studies (93 patients) [18, 20], a bougie of 34 Fr was used in two studies (43 patients) [24, 29], a bougie of 38 Fr was used in two studies (38 patients) [28, 30], a bougie of 40 Fr was used in only one study (25 patients) [27], and in another study the bougie size used was not reported (53 patients) (Table 4) [31]. Oversewing of the staple line was performed in four studies (132 patients) [18–20, 27]. Distance of gastrectomy from the pylorus ranged between 2 and 6 cm (Table 4) [18–33]. The mean preoperative and postoperative BMI ranged between 38.8 and 48.97 kg/m^2^ and 28.2 and 40.7 kg/m^2^, respectively, but postoperative BMI was reported only in eight studies [18–33].

**Table 4:** Pre- and intraoperative results

Author	Preoperative exclusion criteria	Number of patients	Number of women (%)/men (%)	Mean age ± SD (range)	Hiatal hernia/hiatoplasty (diagnosis)	Bougie size (French)	Distance of gastrectomy from pylorus (cm)
Braghetto et al. [18]	GERD symptoms, esophagitis, abnormal manometry	20	17 (85)/3 (15)	37.6 ± 12.6 (23–55)	-	32 oversew	2-3
Gorodner et al. [19]	Erosive esophagitis, Pathological pH-monitoring	14	13 (92.9)/1 (72.1)	42 ± 12	4/0 (barium swallow)	36 oversew	6
Valezi et al. [20]	Previous gastric surgery, history of GERD, esophagitis, hiatal hernia, conditions that could affect esophageal motility	73	55 (75.3)/18 (24.7)	40.2	-	32 oversew	4
De Angulo et al. [21]	-	26	14 (53.8)/12 (46.2)	45.27 ± 10.14	5/1 (endoscopy)	36	5
Sioka et al. [22]	-	18	10 (55.6)/8 (44.4)	40.7 ± 8.1 (30–56)	-	36	5
Rebecchi et al. [23]	Large hiatal hernia, previous gastric surgery	65 ^†^	n.r.	n.r.	11/0 (barium swallow)	36	6
Kleidi et al. [24]	Previous upper gastrointestinal surgery, history of GERD, upper dysmotility, medication for upper gastrointestinal disease	23	11 (47.8)/12 (52.2)	38.5 ± 10.9	-	34	3-4
Gemici et al. [25]	Esophageal motor disorder, advanced stage of hiatal hernia	62	50 (80.6)/12 (19.4)	40.3 ± 10.6 (20–58)	-	36	2-3
Coupaye et al. [26]	Erosive esophagitis. PPI dependency for GERD	30* 47^	46 (97.9)/1 (2.1) ^‡^	41.1 ± 9.4	4/0 (n.r.)	36	5-6
Del Genio et al. [27]	GERD symptoms, previous upper gastrointestinal surgery, hiatal hernia, erosive esophagitis, Barrett esophagus	25	18 (72)/7 (28)	Median 42 (22–62)	-	40 oversew	n.s.
Tolone et al. [28]	Signs of pathological GERD, hiatal hernia, esophagitis, Barret esophagus	26	n.r.	n.r.	-	38	6
Burgerhart et al. [29]	-	20* 15^	16 (80)/4 (20) n.r.	43 ± 12 n.r.	5/0 (manometry)	34	6
Georgia et al. [30]	Erosive esophagitis, use of PPI, GERD symptoms	12	9 (75)/3 (25)	39.67 (SE 1.86) (23–55)	-	38	2-3
Świdnicka-Siergiejko et al. [31]	Hiatal hernia > 3 cm, patients with esophageal manometry abnormalities	53	32 (60.4)/21 (39.6)	Median 41.6 (20 - 70)	-	-	-
Thereaux et al. [32]	Erosive esophagitis,PPI-refractory, GERD	50	39 (78)/11 (22)	n.r.	3/3 (manometry)	96	6
Yormaz et al*.* [33]	Previous thoracic, esophageal or gastric surgery, hiatal hernia, gastrointestinal pathology, or malignancy	152	104 (68.4)/48 (31.6)	41.2 (26–65)	-	36	2 and 6

*PPI* proton pump inhibitor, *GERD* gastroesophageal reflux disease. ^†^: initially 71 patients were included, but 6 patients were lost at follow-up, the number of women/men lost is not reported. *: number of patients evaluated by manometry. ^ number of patients evaluated by pH-monitoring. ^‡^: number of women/men who underwent manometry and/or pH-metry is not reported. *n.r.* not reported, *SD* standard deviation, *SE* standard error

### Esophageal manometry

Fourteen articles out of 16 assessed esophageal motility prior to and after LSG analyzing the lower esophageal sphincter (LES) characteristics and esophageal body peristalsis [18–31]. Conventional manometry was performed in eight studies, [18–25], while in another four studies data were obtained from high-resolution manometry (HRM) [26–29]. Results from these studies are very heterogeneous: use of different techniques, such as conventional manometry or HRM, different parameters analyzed, and variable postoperative manometry timing ranging from 1 to 12 months after surgery [18–31]. Two studies did not report manometric data because manometry was used only to rule out major motility disorders or to identify the position of the LES [30, 31]. The remaining 12 studies assessed LES characteristics and esophageal body peristalsis [18–29] (Table 5).

**Table 5:** Manometric and pH-monitoring results

Author	Timing after surgery (months)	Type of manometry	Postoperative manometric parameters	Manometric results	Type of 24-hours pH monitoring	Postoperative pH-monitoring parameters	pH-monitoring results
Braghetto et al. [18]	6	Conventional	LESP: 10.5 ± 6.06 mmHg (12.3 - 23.9)	LES hypotonia de novo in 85%	-	-	-
Gorodner et al. [19]	12	Conventional	LES length: 3.2 ± 1.3 cm LESP: 12.4 ± 4.5 mmHg NSEMD: 2 IEM: 1 DEA: 75 ± 26 mmHg	Decrease LESP	pH-metry	DMS: 28.4 ± 19 NRE: 25.4 ± 12.8 NRE > 5: 4.7 ± 3.9 LER: 27.3 ± 24.6 TAET: 110 ± 83	Not change: 7% Improvement: 21% De novo: 36%
Valezi et al. [20]	12	Conventional	LESP: 12.6 ± 8.7 mmHg Wave amplitude: 146.5 ± 37.7 mmHg Wave duration: 5.7 ± 1.1 mmHg Normal peristalsis: 90%	Decrease LES pressure LES hypotonia: from 11% to 44% Increase of amplitude contraction wave Peristalsis: from 100% to 90%	-	-	-
De Angulo et al. [21]	12	Conventional	LES length: 4.06 ± 1.21 cm Wave amplitude: 98.38 ± 34.26 mmHg LESP: 13.2 ± 6.19 mmHg	Decrease contraction and LESP	pH-metry	DMS: 37.3 ± 29.77 NRE > 5: 3.92 ± 4.1 NRE: 135.76 ± 97.79 LER: 21.11 ± 21.04 TAET: 113.46 ± 100.13	Improvement: 50% De novo: 66%
Sioka et al. [22]	6-15	Conventional	Wave amplitude: 69.8 ± 26.3 mmHg	Decrease of body contraction, and LESP	-	-	-
Rebecchi et al. [23]	24	Conventional	Group A LES length: 3.1 ± 0.4 cm LESP: 10.1 ± 4.2 mmHg Distal esophageal wave amplitude: 82.8 ± 18.9 mmHg Group B LES length: 4 ± 0.4 cm LESP: 16.4 ± 4.5 mmHg Distal esophageal wave amplitude: 90.6 ± 19.8 mmHg	Not significant change in amplitude and LESP	pH-metry	Group A DMS: 10.6 ± 5.8 TAE: 4.2 ± 2.6 % Group B DMS: 12 ± 2.3 TAE: 3.5 ± 1.1 %	Not change: 14.3% Improvement 85.7% De novo: 18.9%
Kleidi et al. [24]	> 6 weeks	Conventional	Total LES length: 4.1 ± 1 cm Abdominal LES length: 2.4 ± 0.9 cm LESP: 21.1 mmHg Contraction amplitude: 133.3 ± 58.3 mmHg	Increase LESP	-	-	-
Gemici et al. [25]	3	Conventional	LES length: 3.34 ± 0.72 cm (2-5) LESP: 16.6 ± 4.4 mmHg (7-29) Amplitude pressure of the esophagus: 80.8 ± 19.3 mmHg (48-133) LESR: 7.27 ± 3.57 mmHg (0-14) Relaxation time of LES: 6.51 ± 1.96 seconds (1.8-10.7) IGP: 10.95 ± 2.87 mmHg (3-19)	Not significant change in amplitude Decrease LESP	pH-metry	DMS: 33.84 ± 26.55 (0.95–113.65) TAE: 9.84 ± 8.09 % (0.20–31.7) NRE: 65.52 ± 50.29 (1–235.9) NRE > 5: 5.36 ± 5.23 (0–25.4)	Significant increase of DMS
Coupaye et al. [26]	12	HRM	LES length: 27.5 ± 25.6 cm LESP: 17.4 ± 9.7 mmHg DCI: 1124 ± 1211 Normal esophageal contractions: 51 ± 31 Patients with IEM: 15 (50%) IGP: 11.7 ± 3.5 mmHg	Decrease of % of normal peristalsis, increase of patients with IEM, Not significant change in LESP	pH-metry	DMS: 23.8 ± 20.5 TAE: 6.3 ± 5.3 % NRE: 65 ± 51	Healing: 44% Improvement: 19% Worsening: 37% De novo: 53%
Del Genio et al. [27]	13	HRM	Median LES length: 3.8 cm Median LESP: 22 mmHg (7-29) Patients with IEM: 46 % (30-50) Complete bolus transit: 50 % (30-70)	Increase of IEM Not significant change in vLESP	Impedance	Median DMS: 18.2 Median total NRE: 53 Median total acid reflux episodes: 16 Median total non-acid reflux episodes: 36 Median acid postprandial retrograde movements: 8 Median non-acid postprandial retrograde movements: 20 Median bolus clearance time: 34	Increase of DMS
Tolone et al. [28]	12	HRM	Median LESP: 22 mmHg Median LES length: 23 cm Median IRP: 6.3 mmHg Median IGP: 18.8 mmHg	Increase of IGP	Impedance	Raw data not reported	Increase in AET and total number of refluxes
Burgerhart et al. [29]	3	HRM	LESP: 11 ± 7 mmHg DCI: 1537.4 ± 1671.8 IRP4: 2.1 ± 5.2 mmHg IGP: 6.5 ± 4.1 mmHg	Decrease of DCI, LESP, IRP and IGP	Impedance	TAET: 12.4 ± 10.4 % TAE: 37 ± 31.8 Total non-acid reflux episodes: 0.2 ± 0.7 NRE > 5: 5.7 (0–27) LER: 25.4 (1.9– 81)	TAET significantly increase
Georgia et al. [30]	12	-	-	-	Impedance	DMS: 47 TAET: 13.27 % Acid reflux upright distal activity: 61.42 Non-acid reflux upright distal activity: 46.75 Bolus exposure acid time: 4.23 % Bolus exposure non-acid time: 4.29 %	Improvement: 8%Worsening: 80% De novo: 50%
Świdnicka-Siergiejko et al. [31]	10-12	-	-	-	Impedance	Median TAE: 54 Median TAET: 40 Median acid clearance time: 68 Median bolus clearance time: 12	Not change: 70.4% Improvement: 17.8% De novo: 17.8%
Thereaux et al. [32]	6	-	-	-	pH-metry	Group 1 Median DMS: 23.8 TAET: 5.6 % Group 2 Median DMS: 20.6 TAET: 5.9 %	Improvement: 33% De novo: 69%
Yormaz et al. [33]	6-12-24	-	-	-	Impedance	Group A (24-month follow-up) Median DMS: 5.83 (1.74–23.6) TAET: 2.31 % (0.76–6.52) Median NRE > 5: 3.32 (1.4–7.3) Group B (24-month follow-up) Median DMS: 7.29 (2.37–25.1) TAET: 3.57 % (1.52–11.82) Median NRE > 5: 4.27 (2.3–7.5)	Improvement of AET and DMS

Variables are expressed as mean ± standard deviation. *HRM* high-resolution manometry, *LES* low esophageal sphincter, *LESP* low esophageal sphincter pressure, *NSEMD* non-specific esophageal motility disorder, *IEM*: ineffective esophageal motility, *DEA* distal esophageal amplitude, *DMS* DeMeester score, *NRE*, number of reflux episodes, *NRE > 5* number of reflux episodes longer than 5 min, *LER* longest episode of reflux (minutes), *TAET* total acid exposure time, *TAE* total acid exposure, *LESR* residual pressure of low esophageal sphincter, *IGP* intragastric pressure, *DCI* distal contractile integral (mmHg · cm · s), *IEM* ineffective esophageal motility, *IRP* integrated relaxation pressure, *IRP4* integrated relaxation pressure over 4 s, *AET* acid exposure time

#### Conventional manometry

Braghetto et al. collected data from twenty obese patients who underwent conventional manometry before and 6 months after LSG [18]. Data analysis was limited to the LES resting pressure and sphincter length [18]. After surgery, LES pressure significantly decreased from 14.2 ± 5.8 mmHg to 10.5 ± 6.06 mmHg (*p* = 0.01); 17 patients (85%) presented de novo hypotensive LES, defined as resting pressure < 12.1 mmHg [18]. Also, the length of LES was affected; prior to surgery, a total LES length of greater than 3.5 cm was measured in all twenty patients, as compared with only six patients after surgery [18]. Abdominal LES length was less than 1 cm in 14 patients [18].

Another prospective study showed similar LES changes 1 year after surgery [19]. LES resting pressure decreased in 14 patients from 17.1 to 12.4 mmHg (*p* ≤ 0.05), and a hypotensive LES was detected in about 30% of patients as compared to 7% of patients preoperatively [19]. One patient developed ineffective esophageal motility 1 year after surgery, but the changes in the parameters evaluating esophageal body motility were not statistically significantly different after surgery [19].

Valezi et al. also observed changes in both esophageal body motility and LES pressure 1 year after surgery in a series of 73 patients [20]. Comparing pre- and postoperative findings, after surgery there was a statistically significant reduction in LES pressure, wave amplitude, and percentage of patients with normal peristalsis [20].

Another prospective study reported changes in esophageal body contractions in 26 patients [21]. Significant decrease of contraction amplitude in the distal esophagus was detected one year after surgery (*p* = 0.025), even if the values remained in a normal range [21]. A similar result was found in LES pressure that decreased significantly, whereas the mean value remained within the normal range [21].

In a series of 18 patients, Sioka et al. observed that patients with normal peristalsis significantly increased from 47 to 82% after a median time interval of 7 months after surgery [22]. In terms of contraction amplitude, however, the value at the upper border of the LES decreased significantly, but each contraction amplitude value remained within the normal range [22]. Finally, the manometric outcomes of LES did not change significantly [22].

Rebecchi et al. did not find any difference in LES resting pressure and distal esophageal wave amplitude in 65 patients 2 years after surgery [23].

Earlier functional evaluation from 6 weeks to 3 months after surgery was reported by two studies [24, 25]. Kleidi et al. carried out an early evaluation with functional test preoperatively and at least 6 weeks after surgery [24]. Data analysis from 23 patients showed a statistically significant increase of total and abdominal LES length [24]. The contraction amplitude in the lower esophagus decreased and there was an increase in reflux symptoms postoperatively [24]. Another early functional evaluation was performed by Gemici et al. [25]. Sixty-two patients were evaluated 3 months after LSG, and the LES resting pressure significantly decreased from 18.8 to 16.6 mmHg [25], with two patients developing a hypotensive LES [25]. Furthermore, prolonged LES relaxation time after swallowing was detected (*p* = 0.001) [25]. Other manometric findings, as total LES length, residual LES pressure, amplitude pressure of the esophagus, and intragastric pressure did not change at 3 months after LSG [25].

#### High-resolution impedance manometry (HRiM)

Data obtained from HRiM studies showed more changes in esophageal body peristalsis in comparison to LES pressure alterations [26–29] (Table 5). Coupaye et al. demonstrated significant worsening of peristalsis in a series of 30 patients [26]. Both the percentage of normal esophageal contractions within the ten analyzed swallows and the mean distal contractile integral (DCI) were decreased at 1 year after surgery [26]. Ineffective esophageal motility (IEM) was detected in 15 patients after LSG compared with 6 patients showing IEM prior to surgery (*p* = 0.048); the percentage of patients with normal esophageal contractions prior to surgery was 71% compared with 51% observed 1 year after LSG [26]. There were no significant changes in LES resting pressure and esophagogastric junction contractile index (EGJ-CI) [26]. Interestingly, when a patients’ subset-analysis based on the presence of GERD after surgery was performed, statistically significant esophageal motility changes were observed only in patients who developed de novo GERD after surgery [26]. Also, postoperative manometric hiatal hernia tended to be more frequent in patients with de novo GERD (55% vs 9%, *p* = 0.06) [26].

Similar results were reported by Del Genio et al. in a series of 25 patients who underwent HRiM before and after LSG, showing unchanged LES function after LSG [27]. Ineffective esophageal motility was detected in 46% of patients after surgery, as compared with 10% before surgery (*p* = 0.0001), and complete bolus transit rate decreased from 90% pre-surgery to 50% after surgery (*p* = 0.0001) [27].

Tolone et al. assessed the effect of different bariatric surgical techniques on EGJ and esophageal peristalsis in 112 obese patients, 26 of whom underwent LSG [28]. After LSG, there was no significant change in LES resting pressure, EGJ-CI, and integral relaxation pressure (IRP), but both intragastric pressure and gastroesophageal pressure gradient delta of pressure (GEPG Δ*P*) were statistically significantly increased [28]. Unlike the absence of significant changes in LES metrics, esophageal peristalsis seems to be affected more by LSG: after surgery, ineffective esophageal motility waves were observed in 36% of patients [28].

Similar changes on LES metric were observed by Burgerhart et al. in a series of 20 patients [29]. Three months after surgery, statistically significant decrease in LES resting pressure and IRP were detected, from 18.3 to 11 mmHg and from 6.5 to 2.1 mmHg, respectively [29]. Esophageal contractility also seemed affected, with a statistically significant decrease in the DCI value from 2006 to 1537 mmHg·cm·sec [29].

**Fig. 2:**
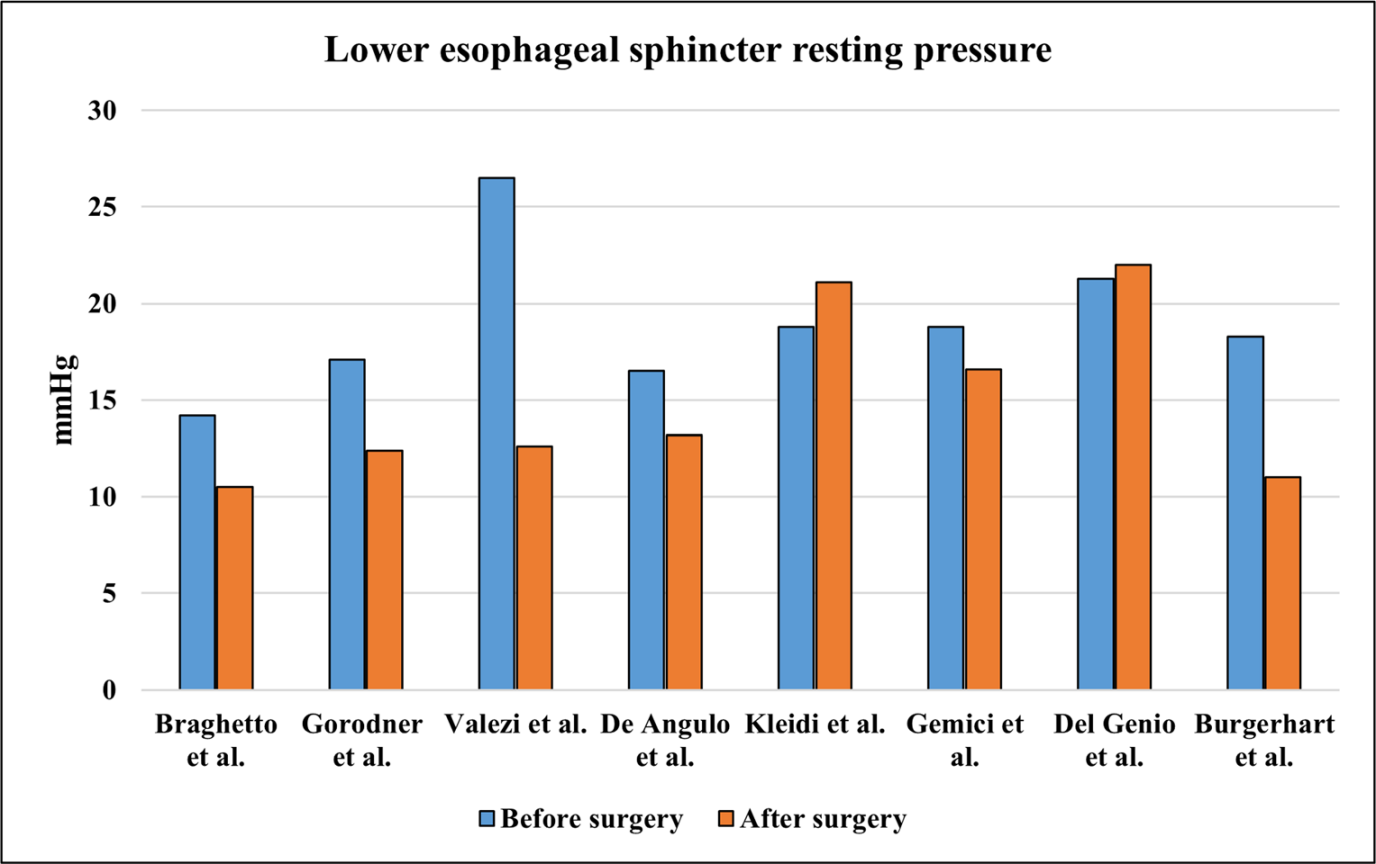
Lower esophageal sphincter resting pressure trend

The LES resting pressures before and after LSG in the included studies are reported in Fig. 2.

### 24-hours pH-monitoring

Twelve studies assessed ambulatory pH-monitoring before and after LSG in order to define its impact on GERD [19, 21, 23, 25–33]. In six studies, 24-hours pH-monitoring was used [19, 21, 23, 25, 26, 32], whereas six more studies characterized GERD by 24-hours impedance-pH monitoring [27–31, 33]. Six studies included patients independently from the presence of GERD [21, 23, 25, 29, 31, 33], whereas the other six articles excluded patients with a preoperative diagnosis of GERD based on the presence of erosive esophagitis, Barrett’s esophagus, typical symptoms, or acid exposure at preoperative pH-monitoring [19, 26–28, 30, 32] (Table 5).

Gorodner et al. observed the most significant changes at pH-monitoring [19]. Increase in DeMeester score (DMS), total acid exposure time (AET), number of episodes longer than 5 min, and duration of longest episode (*p* = 0.05) were detected 1 year after surgery [19]. Compared with preoperative results, GERD worsening and de novo GERD occurred in 21 and 36% of patients, respectively [19].

De Angulo et al. assessed GERD in a population of 26 obese patients who underwent LSG [21]. On reflux monitoring, an improvement of GERD was observed in half of patients, while the de novo GERD rate was 66% [21]. An analysis of each pH-monitoring parameters showed a statistically significant increase in DMS from 24.02 to 37.3 (*p* = 0.028). Other parameters, as total AET and number of refluxes did not change significantly [21].

Another prospective study including 65 consecutive patients showed a significant improvement of pH-monitoring outcome measures after surgery in patients with GERD diagnosed prior to surgery [23]. At baseline, 42.3% of patients had pathologic pH-monitoring; a significant decrease in total AET and in DMS were demonstrated 2 years after surgery, and pathological reflux persisted only in 14.3% of these patients [23]. On the other hand, patients with normal pH-monitoring before surgery (57.7%) developed de novo GERD in 18.9% of cases [23]. Of note, the mean value of DMS and of the total AET did not change significantly after LSG in this group [23].

Gemici et al. evaluated a cohort of 62 obese patients 3 months after surgery, observing the statistically significant increase of several pH-monitoring parameters: DMS, total AET, total number of acid reflux episodes longer than 5 min, and duration of the longest reflux episode [25]. At multivariate analysis, the number of refluxes longer than 5 min was the parameter that influenced most the DMS (odds ratio: 1.866) [25].

Coupaye et al. evaluated a consecutive series of 47 patients before and 1 year after surgery [26]. The study design excluded patients with erosive esophagitis or under proton pump inhibitor (PPI) therapy for GERD [26]. After surgery, significant increase in total AET and in DMS was observed in the entire cohort [26]. However, when patients were stratified based on baseline reflux monitoring, the parameters of reflux changed significantly only in the group of patients without baseline GERD [26]. In this group, DMS increased from 7.5 to 22.3 and total AET increased from 1.8 to 5.8% (*p* = 0.001), whereas no significant increase was observed in patients with baseline GERD [26].

These results do not differ when early functional evaluation was performed [32]. Thereaux et al. observed de novo GERD in a consistent number of patients 6 months after surgery [32]. Fifty patients underwent pH-monitoring before and after surgery [32]. DMS and total AET increased after surgery in patients without preoperative GERD, whereas a significant reduction of the same outcome was detected in GERD patients [32]. In multivariable analysis, lower preoperative percentage of total time spent with esophageal pH < 4 was significantly associated with higher absolute variation after surgery in the percentage of total time spent with esophageal pH < 4, regardless of age, sex, and percentage total weight loss [32].

#### 24-hours impedance-pH monitoring

Interesting findings stemmed from impedance-pH monitoring outcome analysis [27–31, 33] (Table 5). Del Genio et al. reported statistically significant increase in the total number of acid and non-acid reflux episodes in 25 obese patients after LSG at 1 year after surgery, as well as increased acid and non-acid postprandial retrograde movements and esophageal bolus clearance time [27].

Tolone et al. assessed ambulatory impedance-pH monitoring 1 year after surgery in a cohort of 26 patients [28]. The study design included only patients without baseline pathological impedance-pH monitoring. After surgery, significant increase in total AET (*p* = 0.05) and in total number of reflux (*p* = 0.001) was observed [28]. The rate of de novo GERD was not estimated [28].

Burgerhart et al. carried out an evaluation 3 months after LSG in a series of 15 patients and reported an increase in total AET from 4.1 to 12% (*p* = 0.004) [29]. In addition, an increase in the number of long refluxes and in their mean duration was observed [29]. The authors hypothesized that this might be due to decreased acid clearance in the distal esophagus and/or ongoing reflux because of a new reflux episode within an earlier episode (re-reflux), as possible mechanisms of worsening GERD [29].

Georgia et al. reported a statistically significant increase in total AET (from 3.87 to 13.27%, *p* = 0.048) in a series of 12 patients after LSG, and de novo GERD occurred in 50% of patients [30]. According to impedance-pH monitoring, the mean number of non-acid reflux episodes increased, while no differences were reported from the analysis of acid reflux [30]. Furthermore, both bolus exposure acid and non-acid percentage times were significantly increased [30].

Świdnicka-Siergiejko et al. evaluated a cohort of 53 patients who underwent LSG [31]. Based on impedance-pH monitoring, GERD was present in 60.4% of patients before surgery [31]. Outcomes 1 year after surgery showed an improvement in 17.8% of patients with preoperative GERD, and de novo GERD was observed in 17.8% of patients [31]. When the whole population was analyzed regardless of baseline reflux monitoring, only the total number of reflux episodes was significantly reduced, whereas significant changes in the median percentage of esophageal AET, acid clearance time, or bolus clearance time were not observed [31].

Yormaz et al. reported a significant improvement of GERD parameters [33]. The study design included 152 patients assessed by impedance-pH monitoring at 6, 12, and 24 months after surgery [33]. In the whole cohort, DMS, total AET and number of refluxes lasting longer than 5 min decreased significantly at 6 and 12 months, whereas no significant changes were observed at 24 months after surgery [33]. According to the authors, the observed changes depended on the surgical technique that was used [33]. The decrease of GERD parameters was higher when gastric resection started 2 cm from the pylorus rather than 6 cm [33]. The mean DMS decreased from 45.32 preoperatively to 32.27 at 6 months and to 14.42 at 1 year after LSG [33]. Although the improvement was significant, the mean score persisted pathological, and this was observed also in the group with gastric resection starting at 6 cm from the pylorus [33].

**Fig. 3:**
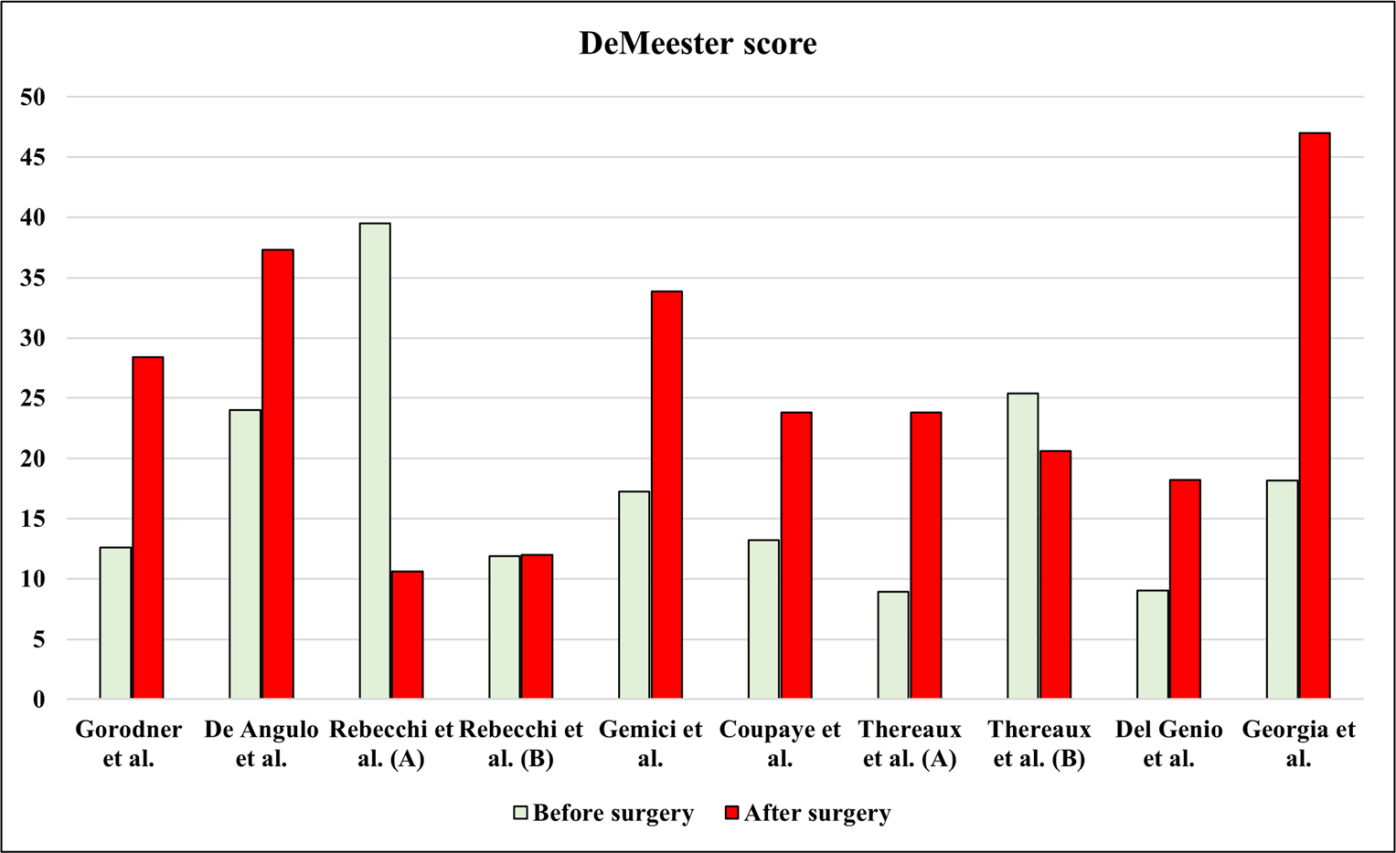
DeMeester score trend

Figure 3 shows the trend in DMSs.

### Symptoms evaluation

Symptoms were evaluated in 11 articles [19, 21–24, 26, 27, 29, 31–33] (Table 6). Eight articles [19, 22–24, 27, 29, 31, 33] employed questionnaires for symptoms evaluation, while the remaining three articles evaluated the presence of symptoms [21, 26, 32]. Overall, in most of the included articles, important modifications of symptoms were not observed [19, 21, 22, 24, 27, 29, 31], worsening of symptoms was reported in two articles [26, 32], and improvement of symptoms was reported in one [23] (Table 6). Symptoms trend seems to be similar to the pH-manometric evaluation in seven articles [22–24, 26, 27, 31, 32], while in the remaining three articles, symptoms are not related to the instrumental data [19, 21, 29].

**Table 6:** SymptomLate esophagogastric anatomic and s evaluation

Author	Symptom assessment tool	Preoperative results	Postoperative results
Braghetto et al. [18]	-	-	-
Gorodner et al. [19]	Standard questionnaire	Heartburn: 0.4 ± 0.9 Regurgitation: 0.5 ± 1 Chest pain: 0.1 ± 0.3 Dysphagia: - Cough: 0.2 ± 0.8	Heartburn: 0.5 ± 1.2 Regurgitation: 0.4 ± 0.6 Chest pain: 0.2 ± 0.8 Dysphagia: - Cough: 0
Valezi et al. [20]	-	-	-
De Angulo et al. [21]	Presence of heartburn and regurgitation	Heartburn: 12 patients Regurgitation: 9 patients	Heartburn: 10 patients Regurgitation: 10 patients
Sioka et al. [22]	Reflux symptoms assessment questionnaire	-	Heartburn: - Improvement: 7 patients - No change: 9 patients - Worsening: 2 patients Regurgitation: - Improvement: 2 patients - No change: 11 patients - Worsening: 5 patients
Rebecchi et al. [23]	Gastroesophageal Reflux Disease Symptom Assessment Scale (GSAS)	Group A: 53.1 ± 10.5 Group B: 45.8 ± 9.6	Group A: 13.1 ± 3.5 Group B: 31.7 ± 15.9
Kleidi et al. [24]	Likert scale	Total score: 4.9 ± 1.6	Total score: 5.8 ± 1.3
Gemici et al. [25]	-	-	-
Coupaye et al. [26]	Presence of heartburn and regurgitation	6 patients	18 patients
Del Genio et al. [27]	Standard questionnaire	-	No modification of symptoms incidence
Tolone et al. [28]	GERDQ questionnaire	n.r.	n.r.
Burgerhart et al*.* [29]	Reflux Disease Questionnaire (RDQ)	GERD score: 7.7 ± 8.3	GERD score: 7.6 ± 6.3
Georgia et al. [30]	-	-	-
Świdnicka-Siergiejko et al. [31]	GERDQ questionnaire	Median 6.5, IQR 6-7	Median 6.7, IQR 6-8
Thereaux et al*.* [32]	Presence of heartburn, regurgitation, or dysphagia	17 patients	29 patients
Yormaz et al. [33]	Reflux Symptom Index (RSI)	n.r.	Group A - 24 months: 25 ± 2.2 Group B - 24 months: 26.4 ± 2.1

Variables are expressed as mean ± standard deviation. *IQR* interquartile range, *GERD* gastroesophageal reflux disease, *n.r.* not reported

## Discussion

The aim of this systematic review was to investigate the manometric and pH-monitoring changes after LSG. Even if the evaluation was heterogeneous among the included studies, overall, it seems that LSG has a negative impact on gastroesophageal reflux evaluated both with manometry and 24-hours pH-monitoring. For a more objective evaluation, we decided to exclude studies in which the analysis was based only on symptoms and on endoscopic findings. In fact, endoscopy may describe only the presence of erosive esophagitis, which is detected only in about 50% of patients with GERD, while 24-hours impedance-pH may also identify non-erosive reflux disease (NERD) with 92% sensitivity rate [34]. Moreover, due to early timing of postoperative endoscopic evaluation reported in the included studies, it is possible that gastroesophageal reflux may not have caused overt damage of the esophageal mucosa yet. For this reason, and for more objective evaluation of the postoperative changes, only studies that reported data of manometry and 24-hours pH-monitoring detected before and after surgery were included.

In our opinion, to define a clear recommendation on the impact of LSG on gastroesophageal reflux, research efforts should be aimed at knowing the position and tone of the EGJ, if there are any modifiable surgical aspects that are involved and if there are any detectable factors prior to surgery that may predict the possible drawbacks of LSG. For this purpose, manometry and 24-hours impedance-pH monitoring are the best instrumental exams that are available [35–38]. Esophageal manometry can detect functional esophageal and EGJ abnormalities which could be involved in the pathophysiology of GERD [35–37]. 24-hours impedance-pH monitoring is the gold standard for detection and characterization of reflux episodes [38]. The impedance-pH monitoring assesses reflux events with both a pH electrode and a series of impedance electrodes [38]. Unlike conventional manometry, this technique provides a more complete evaluation of reflux episodes, which includes weakly acidic and alkaline reflux, liquid gaseous refluxes or both, and re-reflux episodes [38].

The major concern after LSG is the development of GERD, but its pathophysiology and prevalence after surgery are not well understood [39].

The anti-reflux mechanisms involve (i) anatomical structures, such as the EGJ, composed of the intrinsic LES and diaphragmatic crura, the phrenoesophageal ligament, the acute angle of His, and (ii) functional barriers such as the functional efficacy of the EGJ and the esophageal peristalsis clearing the refluxate from the esophageal lumen to the stomach [38].

LSG may modify the anti-reflux mechanisms at different levels resulting in an increased risk of developing GERD [8, 13]. For this reason, in obese patients with complicated GERD, as severe erosive disease or Barrett’s esophagus, LRYGB is usually preferred [1, 9–11, 40]. However, because of the heterogeneity of the available studies, the prevalence of GERD occurring after LSG is still not well defined [39].

Several questions have recently stimulated medical research on this topic: is LSG responsible for GERD? Which alterations can occur after LSG and which alterations can contribute to GERD development? Could surgical technical aspects cause functional and/or anatomical changes, which may be responsible for GERD pathophysiology? Are there any baseline functional abnormalities which are predictors of the development of GERD after LSG?

Studies including functional testing before and after LSG were selected to provide an objective assessment of reflux disease and to try to explain its pathophysiology [18–33]. Data analysis was hampered by a lack of standardization both in re-assessment timing, ranging from 6 weeks to 24 months after surgery, and in the type of outcomes that were evaluated [18–33]. Because of possible anatomical adaptations occurring over time after surgery, it might be incorrect to equate early and late evaluation.

### Esophageal manometry

A functional evaluation of the main components of the anti-reflux mechanisms, as provided by esophageal manometry, could be useful to understand the pathophysiology of GERD [35, 41]. GERD may be associated with hypomotility and structural mechanisms involving both esophageal peristalsis and the EGJ [35]. It must be considered that the normal range values are defined in “healthy” patients who did not undergo surgery [41]; the anatomical implication of LSG creates a new “system,” and standardized normal values might not be valid in this condition. Normal values refer to asymptomatic subjects, free of systemic pathologies that may affect muscle motor activity or neurological control, who have not previously undergone surgery.

Therefore, the data collected at manometry after surgery must include the following: (1) if there is a more or less stable variation of the recorded variables with respect to the normal ranges; (2) if the “different” adjustment of the values is associated with the presence of symptoms and in this case of GERD; and (3) if a cut-off is identifiable with respect to which of the variations may become the cause of symptoms or in any case of prolonged acid exposure or reflux to the esophageal mucosa.

The relationship between GERD and esophageal body peristalsis impairment is not yet well characterized. Whether the alteration of peristalsis is a cause or a consequence of GERD is still debated; more specifically, is not clear if impairment of peristalsis promotes GERD or if GERD, resulting from other injured anti-reflux mechanisms, may translate into worse peristalsis [42].

The association between GERD and esophageal body peristalsis abnormalities has been demonstrated by several studies [43–46]. The burden of reflux disease increases progressively based on the type of esophageal body motility disorders, from fragmented peristalsis, followed by ineffective esophageal motility, and by absent contractility [47]. Rengarajan et al. demonstrated that failed more than weak peristalsis is associated with reflux burden, probably because of more severe dysmotility which affects both primary and secondary peristalsis and consequently reduces the reflux clearance [42]. One study found significant correlations between manometric and impedance-pH-metric outcomes: AET, number of long-term acid reflux episodes, and number of weakly acid reflux episodes were found to be negatively correlated with esophageal body motility [48]. Most of the included studies reported an impaired peristalsis regarding the amplitude of contractions, by conventional manometry, and DCI by HRM [18–33]. This data is more evident in the pre-LES distal portion of the esophageal body. The reduced or ineffective peristalsis could be an adaptation to the pressure reduction of the EGJ, but in this case it becomes a vicious circle because this situation could lead to ineffective clearing and therefore to a prolonged time of acid exposure.

The effectiveness of the anti-reflux barrier, as well as the severity of reflux, is dependent on function or dysfunction of each individual components of the anti-reflux barrier [35]. A complete characterization of the EGJ should include an evaluation of EGJ-CI and of EGJ morphology which may help to discriminate normal from abnormal barrier functions [35]. Although to date standardized calculation methodology and normative value of EGJ-CI are lacking [41], a lower value of EGJ-CI was observed in patients with pathological AET [49]. Furthermore, Gor et al. demonstrated that EGJ-CI discriminated normal from abnormal AET better than conventional LES parameters [50]. Interestingly, a recent study suggests that lower EGJ-CI affects GERD burden especially if an esophageal body motility dysfunction coexists [42]. EGJ morphology also affects effectiveness of the anti-reflux barrier [51, 52]. Based on overlap or separation between the intrinsic LES and the crural diaphragm (CD), three types of EGJ are defined [52]. In type I EGJ, these structures are overlapping, whereas type III consists of 3 cm or greater separation between the intrinsic LES and CD [52]. Type III EGJ is associated with reflux burden [35, 42]. Low LES resting pressure is more likely in patients with erosive reflux disease and in patients who are candidates to reflux surgery [53, 54], suggesting that a hypotensive LES is associated with higher severity of GERD [36, 37].

The impact of LSG on EGJ-CI and EGJ morphology are not yet well studied [18–33]. In our systematic review, only two studies assessed EGJ-CI and EGJ morphology, and significant changes after surgery nor correlation with pathological reflux monitoring were not observed [26, 28]. Further studies, including complete and standardized evaluation of esophageal motility are required.

Table 5 summarizes the main changes detected by esophageal manometry after LSG. Regarding esophageal peristalsis, an increase of IEM diagnosis and a reduction in contraction amplitude or in DCI, as expression of contraction vigor, were demonstrated [21, 22, 24, 26–29]. The IEM rate after LSG was reported to occur between 36 and 50% of cases [26–28]. A decrease in DCI was also found [29], together with a decrease in contraction amplitude measurements when conventional manometry was performed [21, 22, 24].

LSG seems to be associated with impaired esophageal peristalsis [28]. Tolone et al. demonstrated an increase of proximal intragastric pressure and gastro-esophageal pressure gradient after LSG [28]. These changes must be considered when contraction amplitude and LES pressure are assessed after surgery [28]. Furthermore, impedance HRM showed significant worsening of complete bolus transit compared to baseline [28]. These data suggest that although the esophagus encounters a greater gastric obstacle to empty itself, it does not react by increasing the force of contraction, but rather it produces a slowed esophageal transit. At pH-reflux monitoring, an increase of superimposed reflux is detected, and number of refluxes longer than 5 min are the most influencing parameters of the DMS [29]. This suggests that a reduction in the clearance of reflux is the main cause of GERD onset; probably a more vigorous esophageal contraction is needed to clear the reflux.

A decrease in mean LES tone value was observed in seven of twelve studies [18–22, 25, 29]; three of them detected and estimated the de novo hypotensive LES rate [18–20]. This result could be more clinically relevant than the reduction in mean LES tone value, especially when a pathologic lower value is not reached [41]. De novo hypotensive LES rate detection ranged from 25 to 85% [18–20]. LSG could directly affect LES pressure; Braghetto et al. speculated that partial resection of the sling fibers during surgery might be the cause of a hypotensive LES and de novo GERD might be due to this anatomical, and consequently functional, impairment [18].

### 24-hours pH-monitoring

The impact of LSG on esophageal function involves not only the esophageal motility but also the reflux burden [39]. The relationship between pH-monitoring outcomes and manometric findings is difficult to assess [39]. Data from impedance-pH monitoring show an increase in the number of acid and non-acid reflux episodes suggesting anatomical consequences, including decreased compliance as a cause of this [27, 28]. Also, an increase in esophageal bolus clearance time was demonstrated [27]; maybe, in this “new” anatomical condition, more vigorous esophageal peristalsis is needed to contrast reflux.

In nine out of twelve included studies, an increase of DMS and/or total AET was observed after LSG [19, 21, 25–30, 32]. On the contrary, two studies [23, 33] detected a decrease in these outcomes while one study reported no difference in the observed DMS and AET, although a reduction in the number of refluxes was demonstrated [31].

Considering the findings of baseline pH-metry, a decrease in total AET is the only factor that is associated with a significant absolute variation after surgery [22]. This result supports the evidence that patients without GERD are more likely to develop reflux after surgery than patients with baseline GERD to worsen.

De novo GERD is the major drawback that can occur after LSG, probably due to anatomical changes that could affect the anti-reflux barrier [18]. The incidence of symptomatic de novo GERD after LSG ranges between 0 and 34.9% [39]. As shown in Table 5, in this systematic review, the de novo GERD rate is wide and ranges from 17.8 to 69% [18–33]. Patients with baseline GERD after LSG may show worsening, improvement, or healing: the improvement rate ranges from 8 to 85%, and one study showed healing in 44% of patients [26]. Detection of de novo GERD may be clinically more relevant than the worsening of single parameters. The worsening of pH-metric outcomes, as DMS or AET, can be a notable result if clinical and endoscopic parameters worsen, including symptoms or the rate of erosive esophagitis; otherwise, its clinical impact could be irrelevant. The presence of different “mechanisms” involved in reflux disease could explain these findings. De novo GERD could arise from an anti-reflux system injury during surgery, while patients with baseline pathologic reflux monitoring probably had GERD risk factors, which are not affected by LSG. It may be hypothesized that obesity is the main risk factor involved in GERD for this group of patients and that BMI reduction after surgery may improve GERD in some patients, even if this is not confirmed by the present study [18–33].

Compared to restrictive-malabsorptive surgery, as LRYGB, a restrictive surgery as LSG seems to have a higher risk of developing GERD [28]. A recent trial demonstrated a de novo GERD rate of 31.6% after LSG compared to 10.7% after LRYGB (*p* = 0.01) and the need for conversion to LRYGB due to GERD in 9% of patients who underwent LSG [40].

### Symptoms

Overall, symptom evaluation in the included studies departs from instrumental GERD assessment [19, 21–24, 26, 27, 29, 31–33]. Currently, endoscopy and pH-manometry are not routinely recommended according to the American Society for Metabolic & Bariatric Surgery (ASMBS) and the European Association for Endoscopic Surgery (EAES) guidelines in patients candidate for bariatric surgery as well postoperatively [55, 56]. So, the indication for LSG or LRYGB and the evaluation of GERD after surgery are based on what patients report, with low reliability [34, 55–59]. This lack of instrumental evaluation could be responsible for controversial data on GERD after LSG [23, 26, 60, 61]. In our opinion, a standardized instrumental evaluation before and after surgery would clarify the impact of LSG on GERD, leading to offer patients a higher postoperative quality of life and a reduced risk of Barrett’s esophagus.

### Limitations

Due to the heterogeneity of data obtained from the included studies, it was not possible to statistically analyze if bougie size, distance of gastrectomy from the pylorus, and postoperative BMI had an impact on postoperative GERD. Anyway, based on this review, these variables do not seem to influence the results.

The main limitations of the present systematic review are the small sample of patients for each included article, the heterogeneity of data reported, and the poor quality of the included papers. Moreover, the timing for postoperative GERD evaluation is not standardized, as well as the type of instruments and questionnaires used for GERD evaluation. The abovementioned limitations affect the statistical analysis and make a meta-analysis impossible, thereby making it difficult to draw firm conclusions.

## Conclusions

After LSG a worsening of GERD evaluated by instrumental exams was observed, with a high prevalence of de novo GERD, despite a similar worsening of symptoms was not observed. However, to understand the clinical impact of LSG and the burden of GERD over time, further studies with long-term follow-up are necessary. Concerning GERD pathophysiology, some functional/motility alterations involving the esophageal body peristalsis, the EGJ, or both occur after surgery, but the consequences on GERD development are difficult to define. To understand the clinical impact of each esophageal motility change, the anatomical changes that occur after LSG ought to be considered. It is possible to speculate that a new anatomical EGJ formed after LSG, and the new pressure or pressure gradient could influence the reflux pathophysiology differently. On these grounds, the standardized values defined in patients with a physiologic EGJ could not be valid for patients who underwent LSG. More vigorous esophageal contractions and a higher EGJ pressure could be necessary to contrast the reflux.

To our knowledge, to date functional testing seems to be unable to identify predictors for GERD development or which patients will develop GERD after LSG more easily. However, more accurate and standardized assessments of the EGJ with new promising metrics, as EGJ-CI, could add further information to predict GERD development after LSG.
